# Supplementary material for: Defective Patient NK Function Is Reversed by AJ2 Probiotic Bacteria or Addition of Allogeneic Healthy Monocytes
Source: Cells. 2022 Feb 16;11(4):697. doi: 10.3390/cells11040697 (PMC8870139; doi:10.3390/cells11040697)
Supplement: Supplementary file 1 [file cells-11-00697-s001.zip › cells-1605951-supplementary.pdf]

Supplementary data

Defective patient NK function is reversed by AJ2 probiotic bacteria or addition of allogeneic healthy monocytes

Table S1. Characteristics of cancer patients and their respective healthy controls used in the study.Total of 40 patients with 10 different cancer types are shown in the table. The majority of patients are pancreatic cancer patients. Age and sex matched healthy controls were used for these experiments. Approximately equal numbers of males to females for both patient and healthy donors were used.

Cancer type	Counts	Gender
Pancreatic	24	12 M, 12 F
Colon	1	M
Prostate	2	M
Breast	3	F
Osteosarcoma	4	M
Liver	1	M
Glioblastoma	2	F
Lymphoma	1	M
Ovarian	1	F
Uterus	1	F

Table S2. Comparison of healthy individuals' and cancer patients' NK cells. The list of defects reported for the function of cancer patients' NK cells in our previous publications.

NK cells	Healthy individuals	Cancer patients	Ref.
Cytotoxicity against cancer stem cells	High	Low	[1-4]
IFN-γ secretion	High	Low	[1-4]
Osteoclasts-induced expansion	High	Low	[1-4]
Ability to differentiate cancer stem cells	High	Low	[1, 3, 4]
NK cell-mediated ADCC	High	Low	[3]
Monocyte induced NK cells activation	High	Low	Current study

Table S3. Characteristics of cancer stem cells and differentiated tumors. Cancer stem cells and differentiated tumors were characterized based on their surface markers, growth rate, susceptibility to NK cell-mediated killing, interaction with NK cells, and susceptibility to chemotherapy or radiotherapy mediated killing in many of our previous papers.

Characteristics	Cancer stem cells	Differentiated tumors	Ref.
Surface markers	High: CD44<br>Low: CD54, MHC-I, B7H1	Low: CD44<br>High: CD54, MHC-I, B7H1	[2, 5-10]
Growth and expansion	Fast	Slow	[2, 5-7, 9]
Susceptibility to NK cell-mediated cytotoxicity	Susceptible	Resistant	[2, 5-10]
IFN-γ by NK cells	Induce IFN-γ by NK cells	Do not induce IFN-γ by NK cells	[5-8]
Susceptibility to chemotherapy	Resistant	Susceptible	[10-12]
Susceptibility to radiotherapy	Resistant	Susceptible	[11, 12]
In-vivo growth	Grow large tumors *in-vivo*	Grow small tumors *in-vivo*	[2, 10, 12]

Table S4. Lower IFN-γ secretion in cancer patients' NK cells when co-cultured with autologous or allogeneic healthy monocytes. NK cells and monocytes of cancer patients and those of the healthy individuals were isolated from PBMCs as described in Material and Methods section. NK cells and monocytes were treated with IL-2 (1000 U/ml) alone (**A**) or with a combination of IL-2 (1000 U/ml) and anti-CD16 mAbs (3 µg/ml) (**B**) or with a combination of IL-2 (1000 U/ml) and sAJ2 (NK cells:sAJ2, 1:2)) (**C**). A crisscross NK cells and monocyte co-cultures were performed. After 18 hours of co-culture, supernatants were harvested and used in multiplex luminex arrays to measure IFN-γ secretion. A representative experiment is shown in the table.

	Patient			Healthy		
	+ IL-2	+IL-2 +anti-CD16	+IL-2 +sAJ2	+ IL-2	+IL-2 +anti-CD16	+IL-2 +sAJ2
**NK cells**	16.88	18.19	18.19	14.25	17.91	154.23
**NK +Patient Mono**	16.42	19.79	300.06	18.49	23.01	1658
**NK+Healthy Mono**	19.44	29.88	1255	23.01	39.11	2516

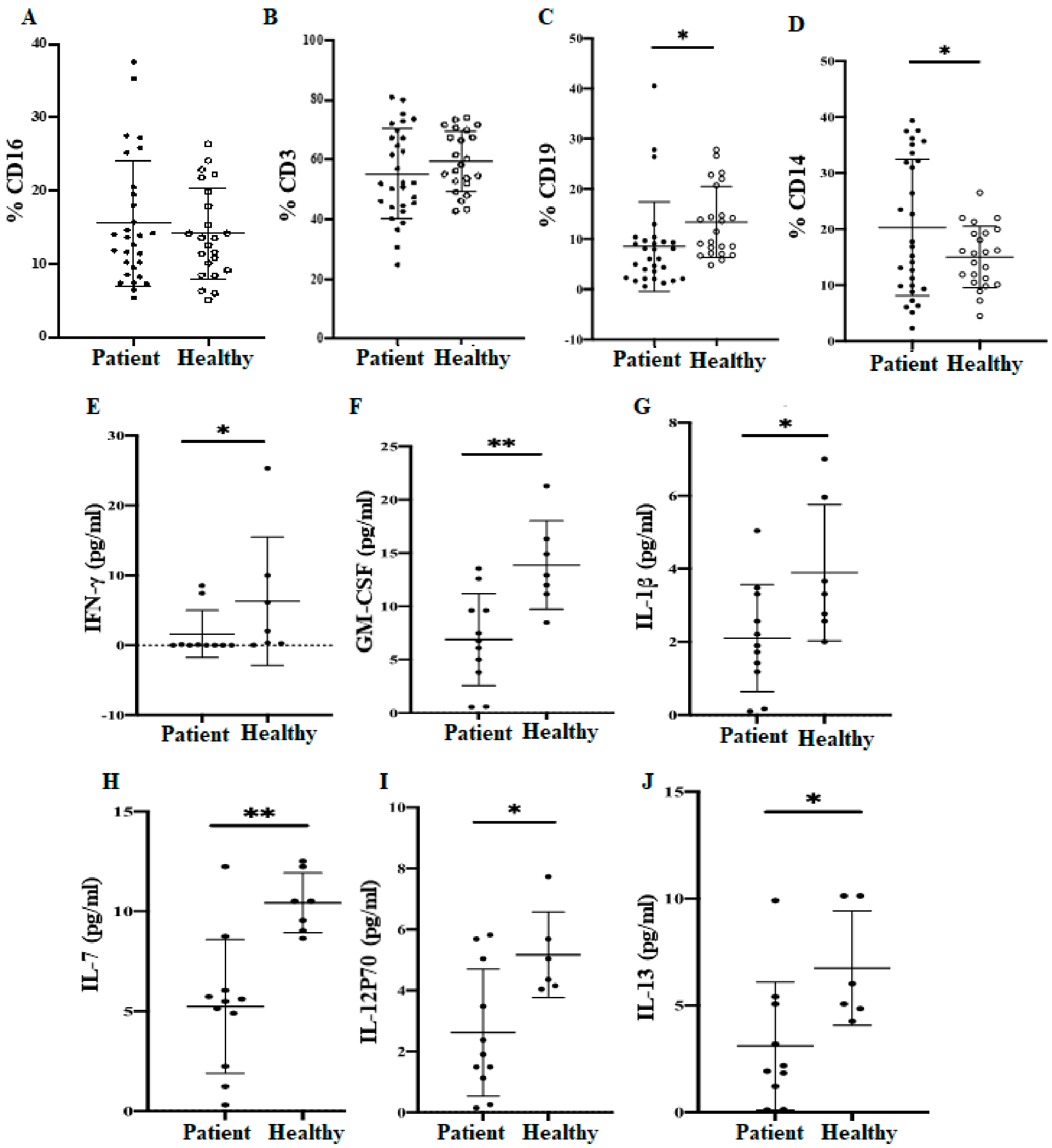

Figure S1. Decreased percentage of CD19+ B cells and increased percentage of CD14+ cells in PBMCs, and decreased cytokines and chemokine secretions in cancer patients' peripheral-derived sera. PBMCs from healthy individuals (n=24) and cancer patients (n=29) were isolated from peripheral blood, and the proportions of mononuclear cells subsets CD16+ (**A**) (n=29), CD3+ (**B**) (n=29), CD19+ (**C**) (n=29) and CD14+ (**D**) (n=29) were determined within CD45+ immune cells using flow cytometric analysis. Sera were collected from the peripheral blood from cancer patients (n=11) and healthy individuals (n=7) and, were analyzed for the levels of IFN-γ (**E**), GM-CSF (**F**), IL-1β (**G**), IL-7 (**H**), IL-12p70 (**I**), and IL-13 (**J**) using a multiplex luminex array. Unpaired t tests were performed for statistical analysis. ****(p value<0.0001), ***(p value <0.001), **(p value 0.001-0.01), *(p value 0.01-0.05).

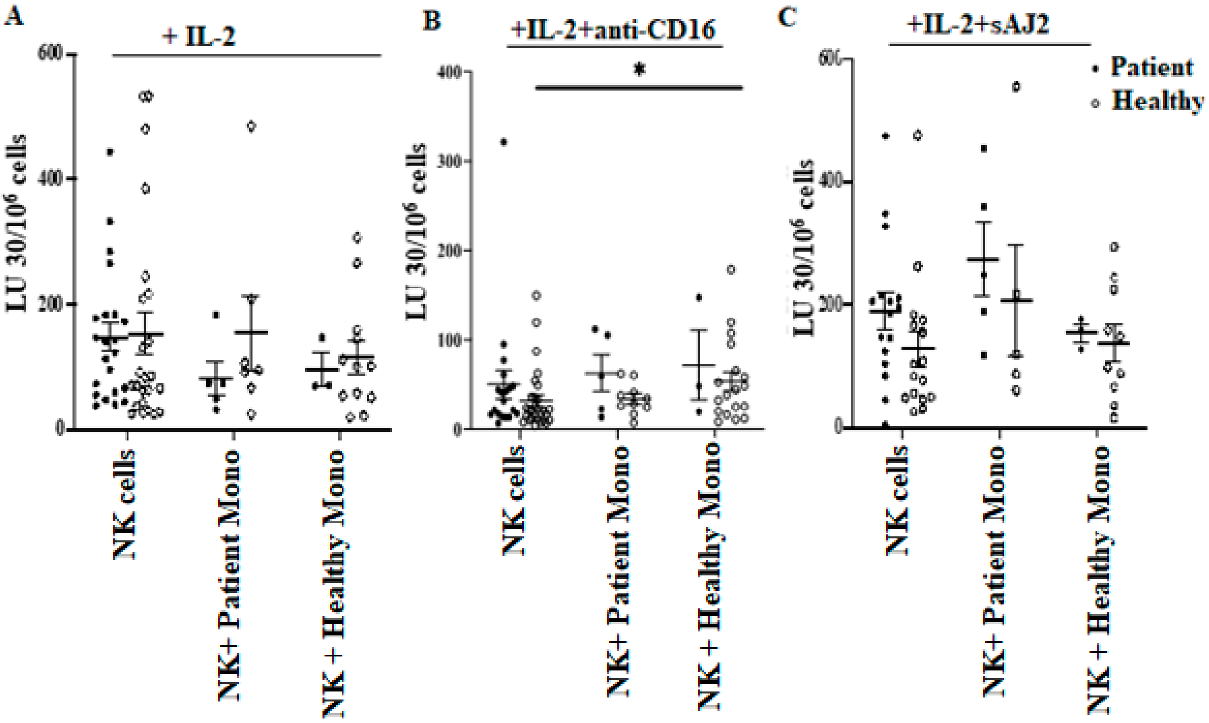

Figure S2. Cancer patients' monocytes suppressed the cytotoxicity in NK cells whereas, increased cytotoxicity in NK cells was seen when they were cultured with healthy individuals' monocytes. NK cells and monocytes of cancer patients and healthy individuals were isolated from PBMCs as described in Material and Methods section. NK cells and monocytes were treated with IL-2 (1000 U/ml) alone (**A**) or with a combination of IL-2 (1000 U/ml) and anti-CD16 mAbs (3 µg/ml) (**B**) or with a combination of IL-2 (1000 U/ml) and sAJ2 (NK:sAJ2, 1:2)) (**C**). A crisscross NK cells and monocyte co-cultures were performed. NK cell mediated cytotoxicity were measured after 18 hours of co-culture using standard 4-hour ^{51}Cr release assay against OSCSCs. The lytic units (LU) 30/10^{6} cells were determined using inverse number of NK cells needed to lyse 30% of target cells OSCSCs ×100. Compiled data are shown in **(A-C)** (n=3 to 29) and data are presented as Mean±SEM. Student t tests were performed to determine statistical significance. ****(p value<0.0001), ***(p value <0.001), **(p value 0.001-0.01), *(p value 0.01-0.05).

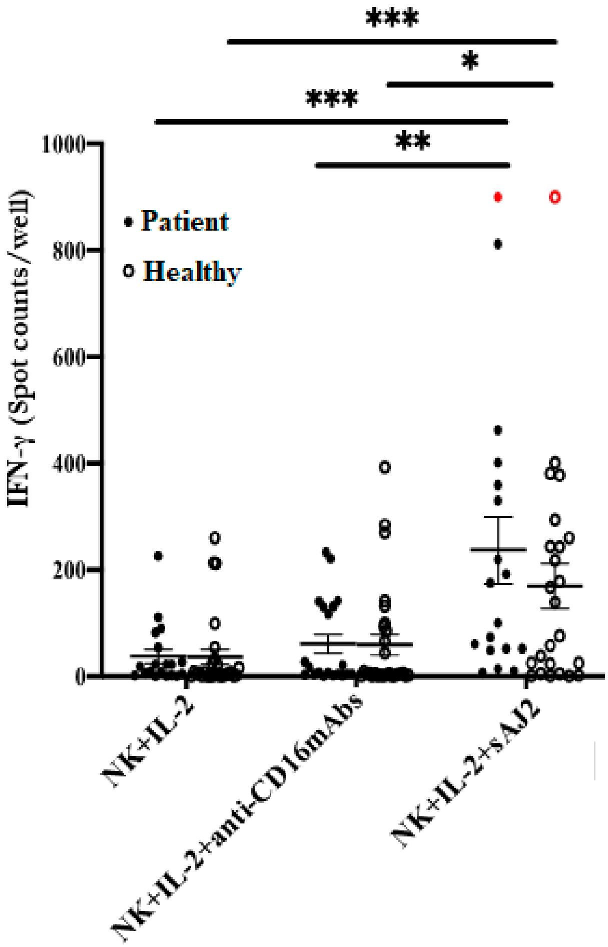

Figure S3. Cancer patients' NK cells exhibited lower IFN-γ secretion when compared to healthy individuals' NK cells. Freshly purified NK cells (1x10^6 cells/ml) from cancer patients and healthy individuals were treated with IL-2 (1000 U/ml) (n=27), with the combination of IL-2 (1000 U/ml) and anti-CD16mAb (3 µg/ml) (n=28), and, with the combination of IL-2 (1000 U/ml) and probiotic bacteria sAJ2 (n=24) at a ratio of 1:2 (NK:sAJ2) for 18 hours before ELISPOT assay was conducted to determine the numbers of IFN-γ spots. ****(p value<0.0001), ***(p value <0.001), **(p value 0.001-0.01), *(p value 0.01-0.05).

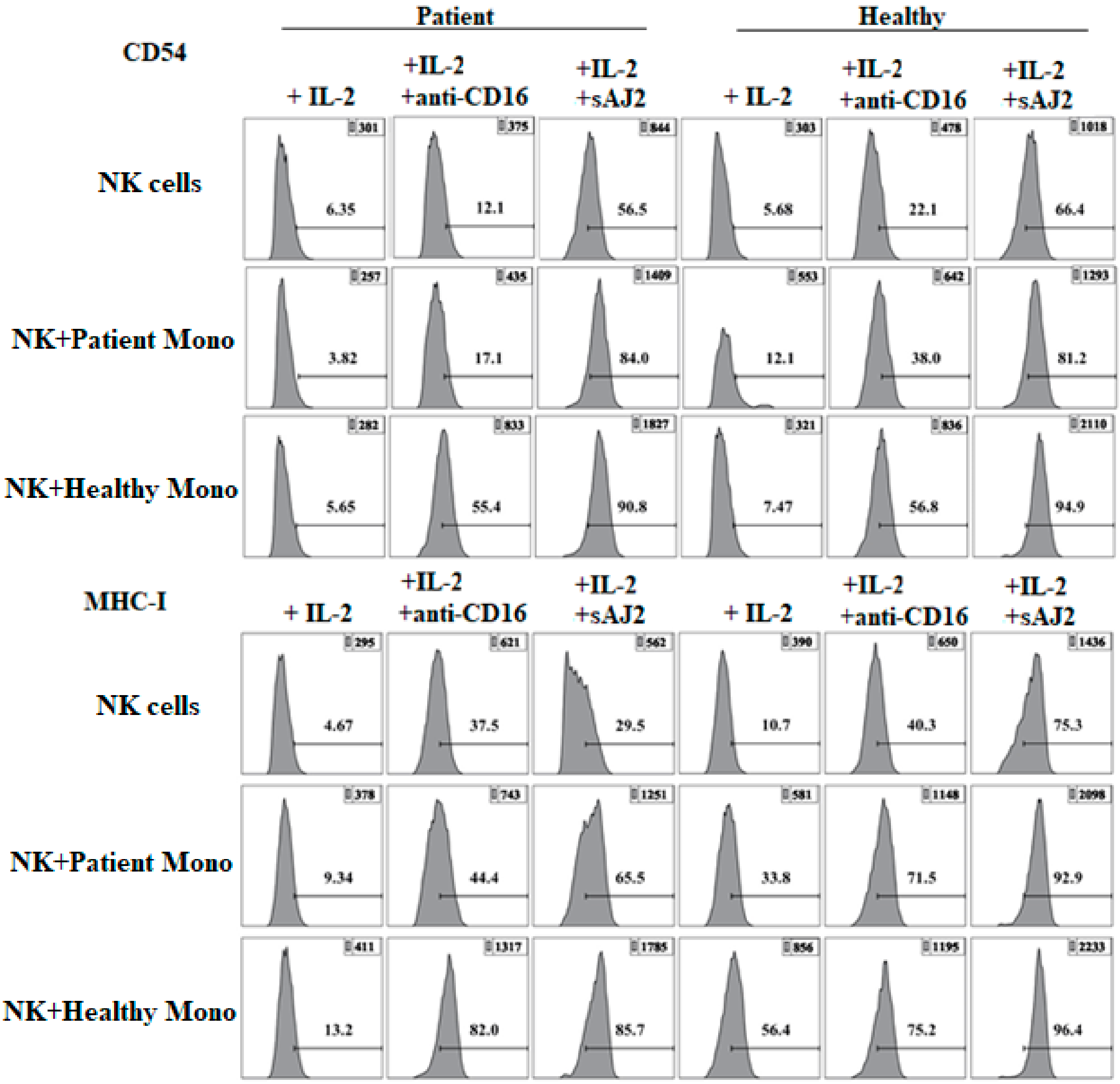

Figure S4. Supernatants harvested from autologous cancer patients' NK and monocytes cocultures treated with IL-2+sAJ2 induced higher differentiation of OSCSCs in comparison to those treated with IL-2 alone or IL-2+anti-CD16 mAbs. OSCSC differentiation assay were conducted as described in Material and Methods section using the supernatants collected from NK cell and monocyte crisscross coculture experiments. Surface expressions of CD54 **(A)** and MHC-class I **(B)** on supernatant treated OSCSCs were determined using flow cytometry. One of the two representative experiments is shown in the figure.

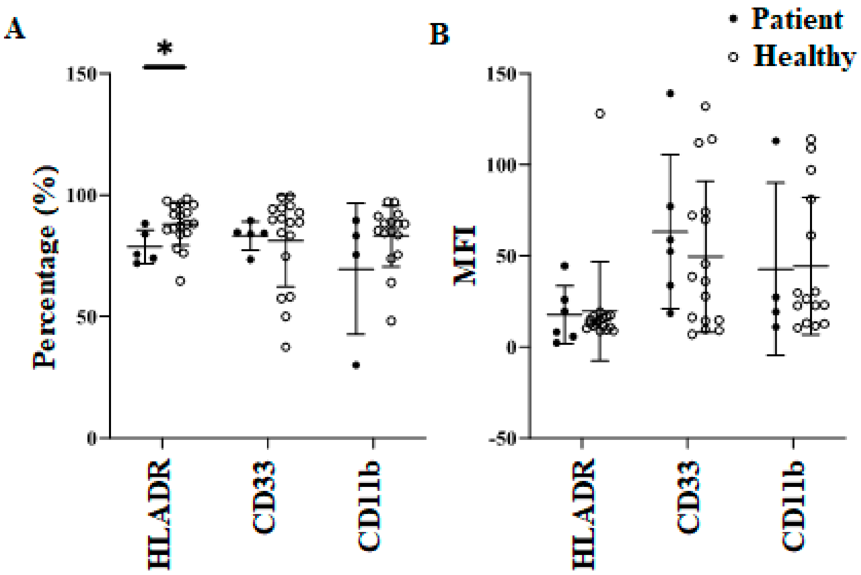

Figure S5. Surface expressions of HLADR, CD33, and CD11b on cancer patients' and healthy individuals' Monocytes. Monocytes of cancer patients (n=6) and healthy individuals (n=14) were isolated from PBMCs as described in Material and Methods section. The surface expression of HLADR, CD33, and CD11b within CD14+ population were determined using flow cytometry after staining with the respective antibodies. Percentage positive cells and mean fluorescence intensity were determined for each surface analysis.

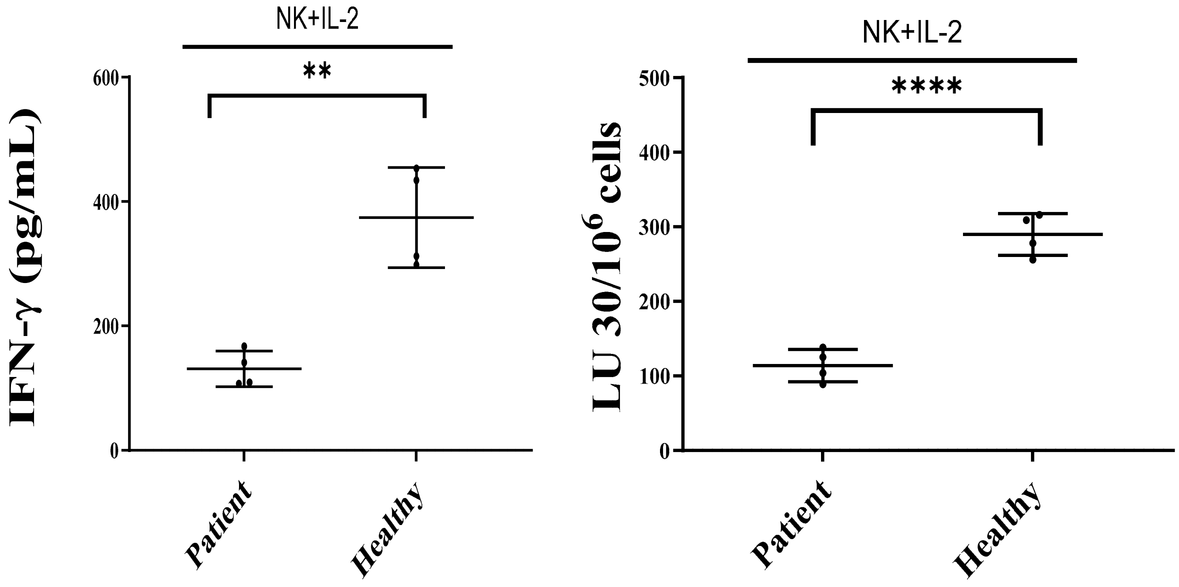

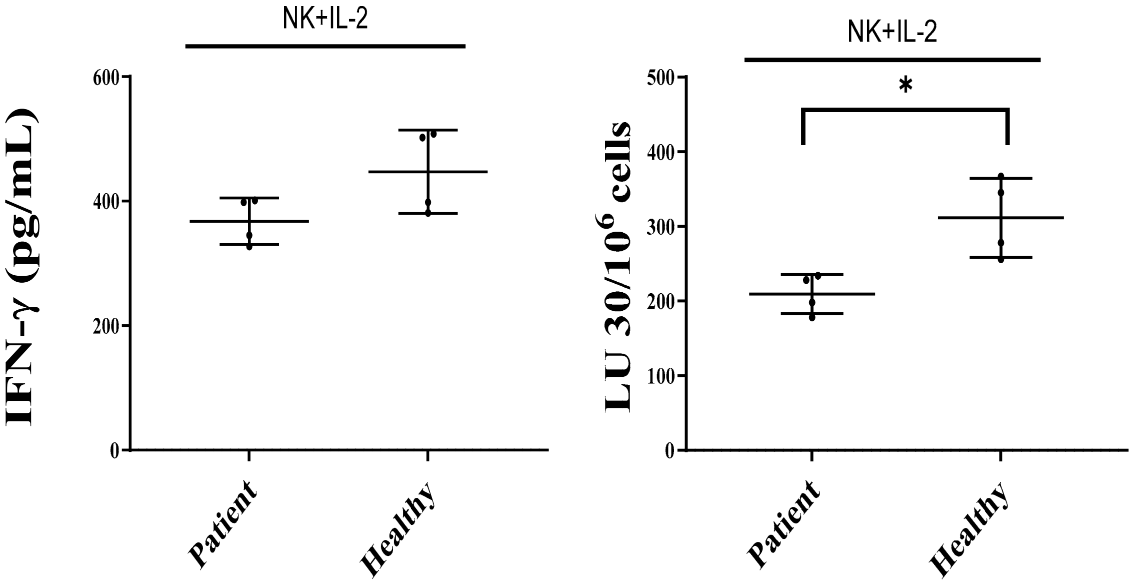

Figure S6. Increased IFN-γ secretion and cytotoxicity in cancer patients' NK cells after oral AJ2 supplementation. Freshly purified NK cells ($1x10^6$ cells/ml) from cancer patients and the healthy individuals were treated with IL-2 (1000 U/ml) for 18 hours before the supernatants were harvested to determine IFN-γ secretion levels using single ELISA (n=4) **(A)**. Freshly purified NK cells ($1x10^6$ cells/ml) from cancer patient and healthy individuals were treated with IL-2 (1000 U/ml) for 18 hours before they were used as effectors to determine cytotoxicity against OSCSCs using standard 4-hour ^{51}Cr release assay. The LU30 were determined as described in Fig. S2 (n=4) **(B)**. Cancer patients were on oral supplementation of AJ2 for four weeks before we analyzed their NK cells. NK cells were treated as described in Fig. S6A, before the supernatants were harvested to determine IFN-γ secretion using single ELISA (n=4) **(C)**. NK cells were treated as described in Fig. S6B, before they were used as effectors to determine cytotoxicity against OSCSCs using standard 4-hour ^{51}Cr release assay. The LU30 were determined as described in Fig. S2 (n=4) **(D)**. ****(p value<0.0001), ***(p value <0.001), **(p value 0.001-0.01), *(p value 0.01-0.05).

References

1. Kaur, K., et al., *Novel Strategy to Expand Super-Charged NK Cells with Significant Potential to Lyse and Differentiate Cancer Stem Cells: Differences in NK Expansion and Function between Healthy and Cancer Patients.* Front Immunol, 2017. **8**: p. 297.
2. Kaur, K., et al., *Probiotic-Treated Super-Charged NK Cells Efficiently Clear Poorly Differentiated Pancreatic Tumors in Hu-BLT Mice.* Cancers (Basel), 2019. **12**(1).
3. Kaur, K., et al., *ADCC against MICA/B Is Mediated against Differentiated Oral and Pancreatic and Not Stem-Like/Poorly Differentiated Tumors by the NK Cells; Loss in Cancer Patients due to Down-Modulation of CD16 Receptor.* Cancers (Basel), 2021. **13**(2).
4. Kaur, K., et al., *Osteoclast-expanded super-charged NK-cells preferentially select and expand CD8+ T cells.* Sci Rep, 2020. **10**(1): p. 20363.
5. Tseng, H.C., et al., *Increased lysis of stem cells but not their differentiated cells by natural killer cells; de-differentiation or reprogramming activates NK cells.* PLoS One, 2010. **5**(7): p. e11590.
6. Jewett, A. and H.C. Tseng, *Tumor induced inactivation of natural killer cell cytotoxic function; implication in growth, expansion and differentiation of cancer stem cells.* J Cancer, 2011. **2**: p. 443-57.

7. Tseng, H.C., N. Cacalano, and A. Jewett, *Split anergized Natural Killer cells halt inflammation by inducing stem cell differentiation, resistance to NK cell cytotoxicity and prevention of cytokine and chemokine secretion.* Oncotarget, 2015. **6**(11): p. 8947-59.

8. Bui, V.T., et al., *Augmented IFN-γ and TNF-α Induced by Probiotic Bacteria in NK Cells Mediate Differentiation of Stem-Like Tumors Leading to Inhibition of Tumor Growth and Reduction in Inflammatory Cytokine Release; Regulation by IL-10.* Front Immunol, 2015. **6**: p. 576.

9. Tseng, H.C., et al., *Differential Targeting of Stem Cells and Differentiated Glioblastomas by NK Cells.* J Cancer, 2015. **6**(9): p. 866-76.

10. Kaur, K., et al., *Super-charged NK cells inhibit growth and progression of stem-like/poorly differentiated oral tumors in vivo in humanized BLT mice; effect on tumor differentiation and response to chemotherapeutic drugs.* Oncoimmunology, 2018. **7**(5): p. e1426518.

11. Kozlowska, A.K., et al., *Differentiation by NK cells is a prerequisite for effective targeting of cancer stem cells/poorly differentiated tumors by chemopreventive and chemotherapeutic drugs.* J Cancer, 2017. **8**(4): p. 537-554.

12. Jewett, A., et al., *NK cells shape pancreatic and oral tumor microenvironments; role in inhibition of tumor growth and metastasis.* Semin Cancer Biol, 2018. **53**: p. 178-188.
